# Supplementary material for: Transformation from crystalline precursor to perovskite in PbCl2-derived MAPbI3
Source: Nat Commun. 2018 Aug 27;9:3458. doi: 10.1038/s41467-018-05937-4 (PMC6110813; doi:10.1038/s41467-018-05937-4)
Supplement: Supplementary file 1 — Supplementary Information [file 41467_2018_5937_MOESM1_ESM.pdf]

Supplementary Information for

Transformation Kinetics from a One-Dimensional Crystalline Precursor to PbCl2 Derived MAPbI3

Stone et al.

TABLE OF CONTENTS

Supplementary Methods 2
Structural Solution and Refinement 2
Extended X-ray Fine Structure (EXAFS) fitting 3
In-situ Grazing-Incidence X-Ray Diffraction (GIXRD) 5
In-situ X-ray Fluorescence (XRF) 6
Activation Energy of Perovskite transformation 8
Kinetics of Cl evaporation 9
Supplementary Note 1: Distinct textures in the precursor film 10
References 11

SUPPLEMENTARY METHODS

Structural Solution and Refinement

The lattice and structure of the precursor MA_2PbI_3Cl phase was determined from grazing incidence x-ray diffraction data. The data was collected at SSRL BL11-3, X-rays of wavelength 0.9744Å were incident on the sample film at an angle of 3° and detected using a MAR Image Plate area detector. The diffraction image was calibrated using a sample of LaB_6 and integrated using the software GSAS-II[1]. The unit cell was assumed to be orthorhombic from inspection of the observed layer lines and the *c*-axis parameter determined directly from their spacing. The indexing of the diffraction pattern was completed using the indexing routing in TOPAS-Academic, confirming a *c*-centered orthorhombic lattice[2].

For structure solution, each layer line of the primary orientation of the MA_2PbI_3Cl phase was integrated independently. These integrated patterns were co-refined, greatly reducing the peak overlap in the data. The structure was modeled in space group *C222* (the lowest symmetry space group of the observed extinction class) using the simulated annealing routine in TOPAS-Academic with a model of independent atoms with variable occupancy. Repeated simulated annealing runs, as well as charge flipping attempts in TOPAS-Academic, converged on a model with four corner-sharing octahedral forming infinite chains along the *c*-axis. This model was refined to determine the final atomic positions and occupancies. Due to the limited data, and the limited scattering power of the methylammonium molecule, only the Pb and halide positions were refined with a fixed thermal parameter for all atoms.

The refined structure exhibits an ordering of the halide atoms such that the corner shared sites (*I2*, *I3*, *I5*) appear to be mixed Cl and I while the exposed sites (*I1* and *I4*) consist primarily of I atoms (site labels are shown in Supplementary Figure 1). The best model gives two distinct sites in the axial direction (*I3* and *I5*) which are symmetry equivalent but with distinct bond lengths corresponding to Pb-I and Pb-Cl bonds, while the equatorial positions (*I2*) are not distinct for Cl and I atoms, but rather occupationally disordered. The mixed halide sites refine to an occupancy which would correspond to roughly 50:50 I:Cl. It should be noted that the diffraction data which could be obtained is limited, and so the details of this refinement should be considered cautiously.

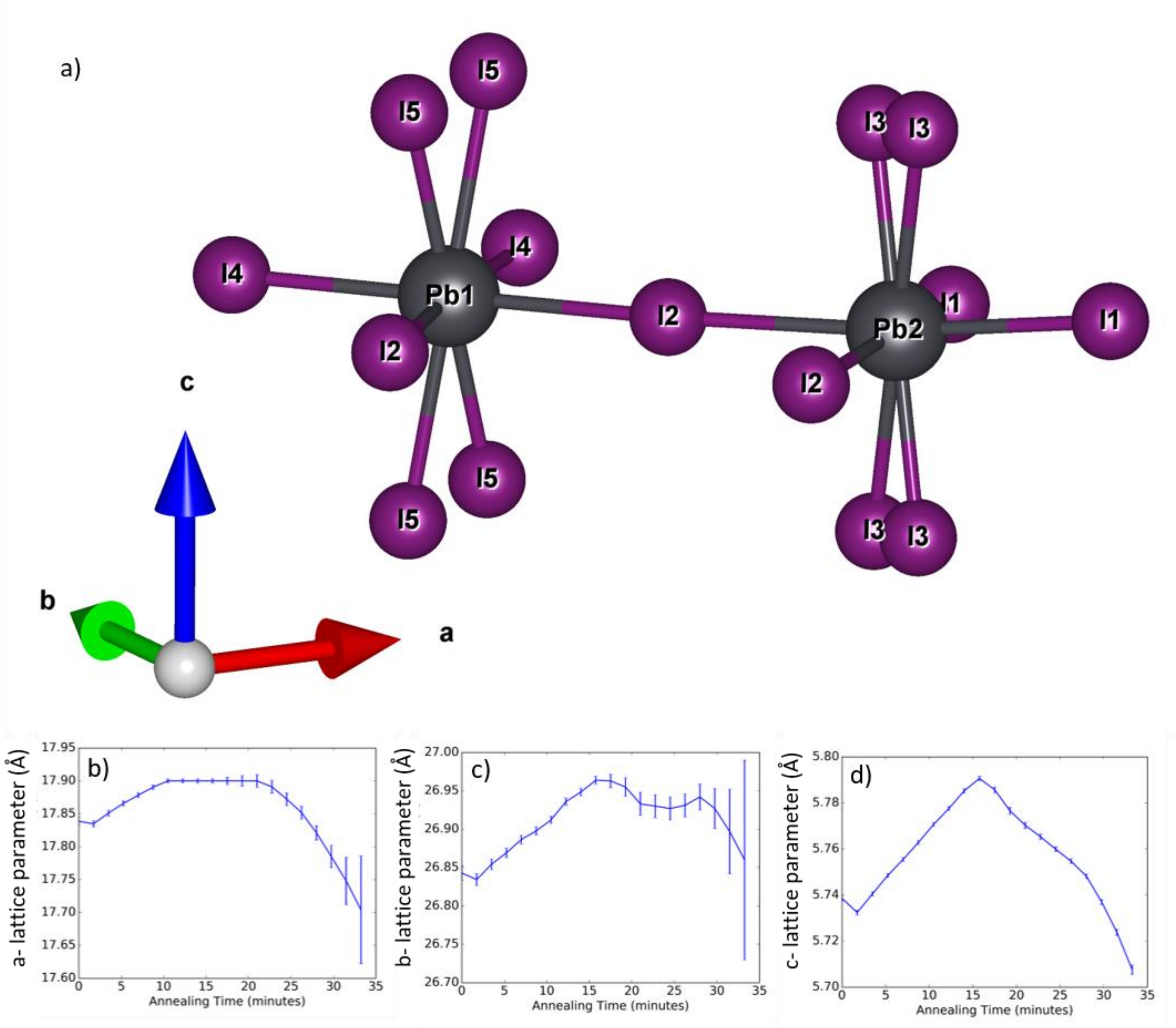

Supplementary Figure 1: Precursor structure and evolution and evolution of lattice parameters during annealing. (a) Structure of MA_2PbI_3Cl showing two corner shared octahedra. The sites are labeled to facilitate discussion of the structure. (b-d) Precursor lattice parameters taken from refinement during annealing of precursor showing evolution of unit cell dimensions.

Extended X-ray Fine Structure (EXAFS) fitting

EXAFS data were measured on a PbI_2 standard and fit in order to fix a value for the amplitude reduction factor S_0^2. PbI_2 was dissolved in DMF and spin-cast onto a glass film. EXAFS were also measured on a precursor film, deposited on glass as described in Methods. The samples were measured the Pb-L_3 edge in a He environment, with fluorescence data measured with a Lytle detector as described in Methods.

The Artemis package was used to subtract the background and fit a post-edge spline.[3] The extracted absorption data were Fourier transformed to real space between k of 3.31 to 12.54 Å^{-1} with a window round dk of 1 Å^{-1}. To remove background noise in the Fourier transform, we fit the spline function using the parameter Rbkg = 1.6 Å, less than half of the Pb-I bond length of 3.228 Å in the PbI_2 structure. First-shell EXAFS were fit from 1.7 to 3.35 Å, using a k-weight of 2. In fitting PbI_2, the degeneracy of the Pb-I path was set to 6, corresponding to the structure.[4] Amplitude reduction factor S_0^2, energy shift ΔE, path length adjustment ΔR, and mean-square displacement σ^2 were allowed to float. The fit is plotted in Supplementary Figure 2 and the fit parameters are listed in Supplementary Table 1. S_0^2 was measured to be 0.85; this value was fixed in the subsequent fits of the precursor EXAFS. Moving onto the precursor,

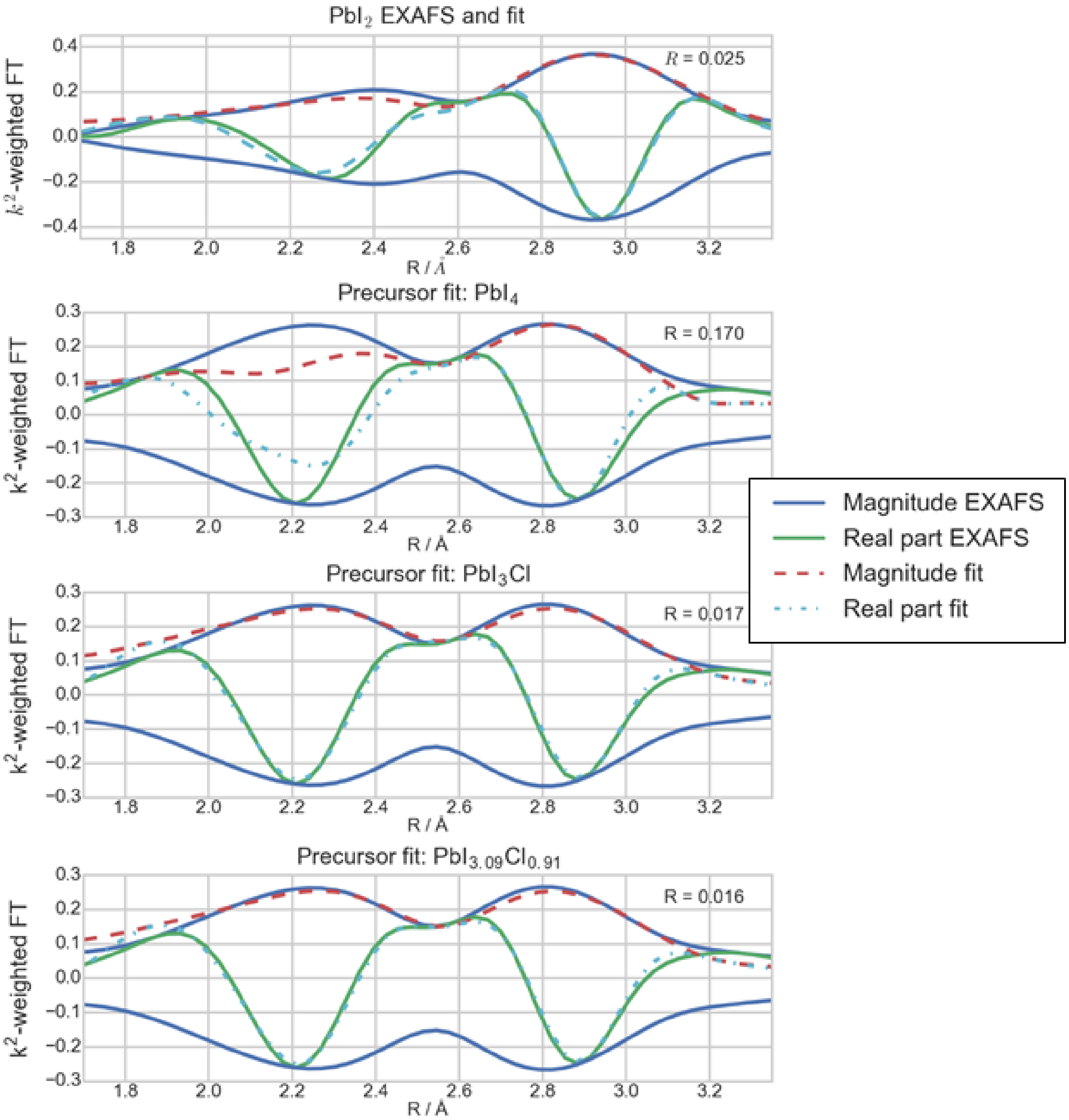

Supplementary Figure 2*: EXAFS fits for PbI_2 and precursor. Blue envelope is the magnitude of the measured EXAFS; green oscillating function is the real part. Red and teal dashed lines are the EXAFS fits.*

we fixed $S_0^2 = 0.85$. Absorption data was collected and processed identically, except for using Rbkg of 1.45 (corresponding to half the Pb-Cl bond length of 2.9 Å). Four different paths were fit to the first shell EXAFS: I and Cl at distances of 2.89 and 3.11 Å, corresponding to the axial and equatorial bond lengths in the refined precursor structure. These 4 values of N: $N_{I,axial}$, $N_{I,equatorial}$, $N_{Cl,axial}$, and $N_{Cl,equatorial}$, were fixed for the fits to PbI_4 and PbI_3Cl and allowed to float for the final fit, as described in Supplementary Table 2. When fit, N was constrained to require 4 equatorial and 2 axial halogens. We recall that both axial, and 2 of the 4 equatorial sites are shared between two octahedra, leading to the reported compositions. The fits are plotted in Supplementary Figure 2 and the fit parameters are reported in Supplementary Table 1.

	PbI_2		*PbI_4*	*PbI_3Cl*	*$PbI_{3.09}Cl_{0.91}$*
**S_0^2**	0.8548 +/-0.1072	**S_0^2**	0.85	0.85	0.85
**ΔE**	-2.2629 +/-1.0040	**ΔE**	-9.8985 +/- 3.0392	-5.2585 +/- 1.2666	-5.7371 +/- 1.7057
**ΔR**	-0.0692 +/-0.0128	**ΔR axial**	-0.0412 +/- 0.0457	-0.0440 +/- 0.0227	-0.0482 +/- 0.0243
**σ^2**	0.0158 +/-0.0015	**ΔR equatorial**	-0.0739 +/- 0.0339	-0.0338 +/- 0.0166	-0.0397 +/- 0.0217
		**σ^2 Cl**	N/A	0.0181 +/- 0.0023	0.0167 +/- 0.0041
		**σ^2 I**	0.0127 +/- 0.0021	0.0165 +/- 0.0011	0.0160 +/- 0.0016

Supplementary Table 1: EXAFS parameters from fits to PbI_2 and precursor film

	PbI_4	PbI_3Cl	$PbI_{3.09}Cl_{0.91}$
N - I,axial	**2**	**0**	*0.19*
N - Cl,axial	**0**	**2**	*1.81*
N - I,equatorial	**4**	**4**	*4*
N- Cl,equatorial	**0**	**0**	*0*

Supplementary Table 2: Path degeneracy values for the precursor fits. Bold values were fixed. Italic values were allowed to float, with the constraint of 2 total axial and 4 total equatorial halide atoms.

In-situ Grazing-Incidence X-Ray Diffraction (GIXRD)

2D GIXRD scans were collected at SSRL beamline 11-3 (Supplementary Figure 3) and integrated to 1D (Supplementary Figure 3b) as described in Methods. The region around the cubic (100) peak at Q=1.0 $Å^{-1}$ was isolated in a Q-range from 0.95-1.05 $Å^{-1}$ and fit to a linear background plus a Voigt profile (Supplementary Figure 3c). The integrated intensity of the Voigt corresponds to the relative amount of crystalline perovskite in the film and is plotted in Figure 4 of the main paper as "perovskite (XRD)."

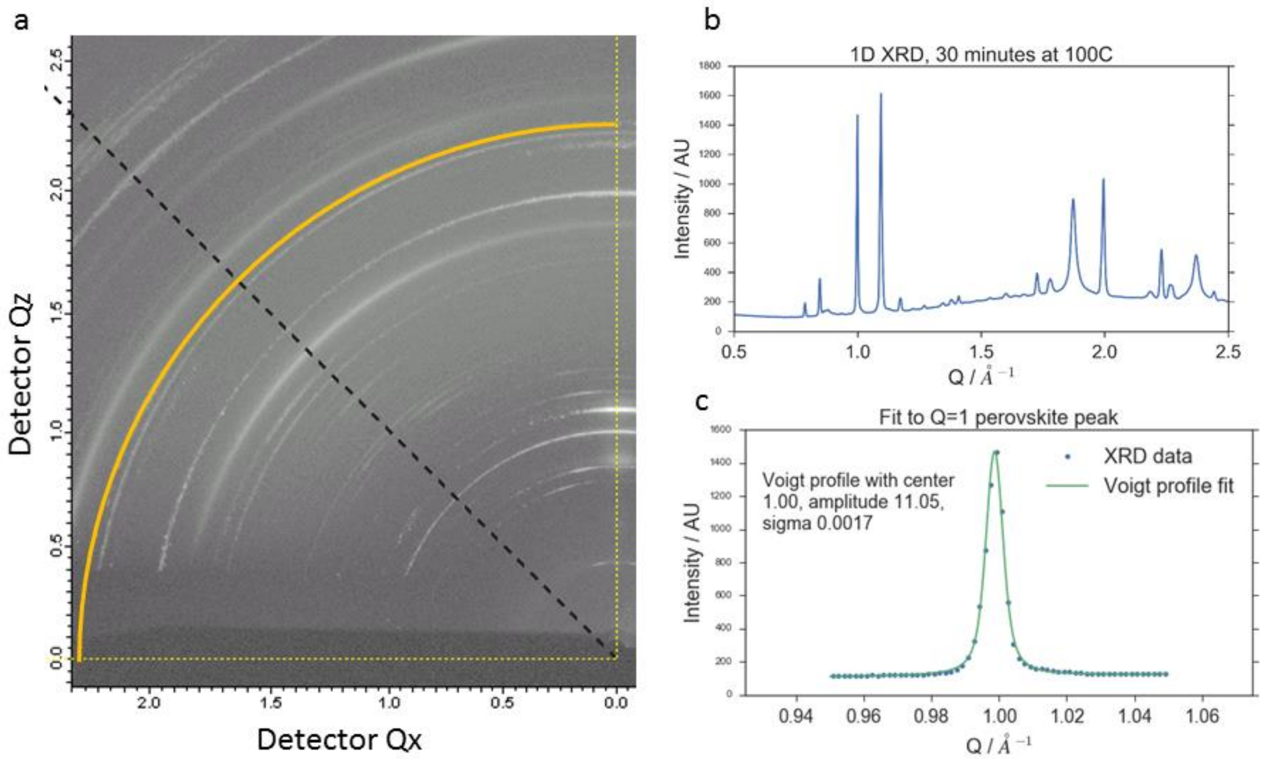

Supplementary Figure 3: Integration and peak fitting of GIXRD data. (a) 2D GIXRD measured on 11-3 at 30 minutes of annealing at 100°C. Region of integration is indicated. (b) The same data, azimuthally integrated to 1D XRD. (c) Voigt profile fit to the perovskite peak at Q=1.

In-situ X-ray Fluorescence (XRF)

The as-spun film was loaded into the sample chamber without air exposure. XRF was measured at SSRL beamline 4-3 using monochromatic X-rays at 3 keV. Fluorescence from the sample was monitored with a Vortex silicon drift detector, which measures the energy of fluorescent photons. As the film was annealed, fluorescence was collected continuously and binned every 30 seconds. Selected XRF spectra for the 95°C anneal are shown in Supplementary Figure 4.

The peak labeled “Pb” is a combination of fluorescence lines M_α (2342.3 eV) and M_β (2444.3 eV). This peak was fit to a pair of Gaussian profiles centered at these energies. The peak labeled “Cl” is a combination of fluorescence lines $K_{\alpha 1}$ (2622 eV) and $K_{\alpha 2}$ (2620 eV), which were treated as a single fluorescence peak. The intensity of the Cl peak is seen to decline with annealing time.

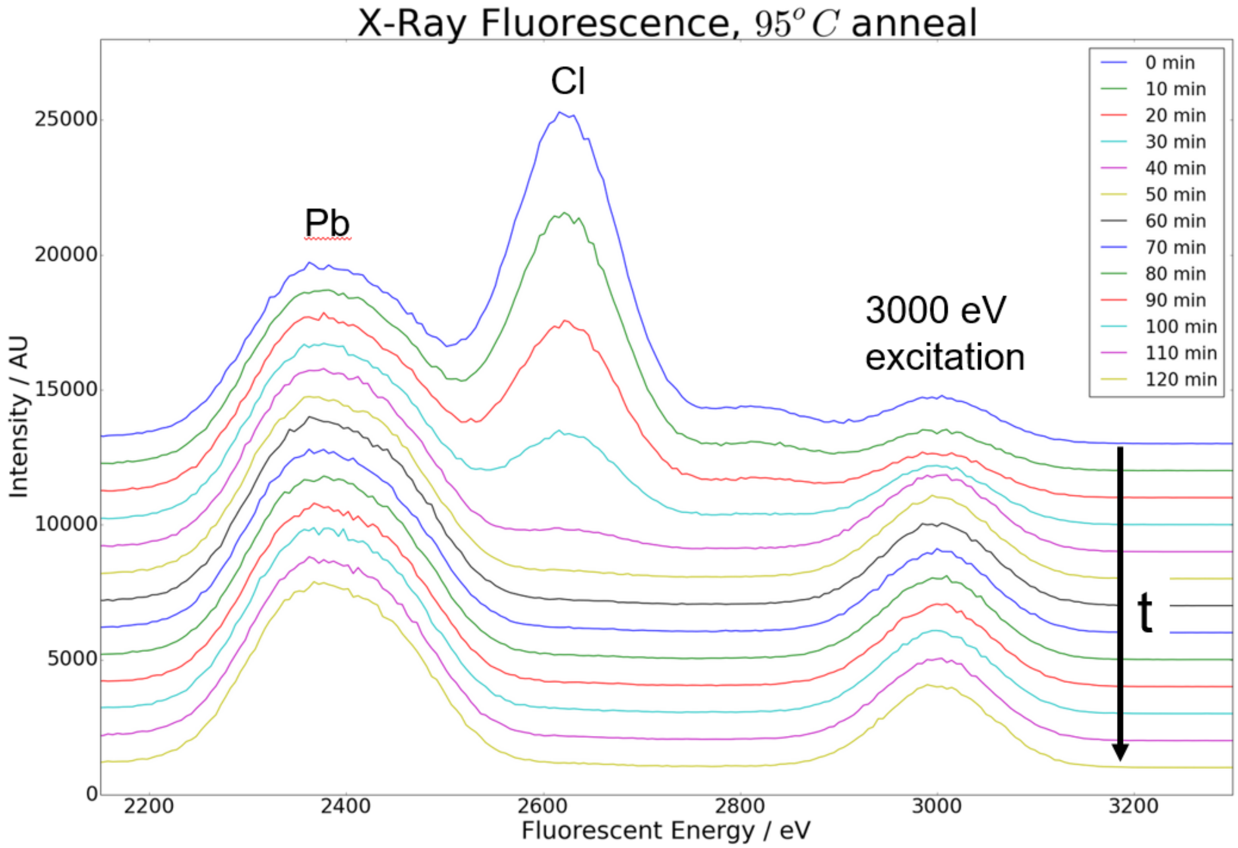

Supplementary Figure 4: In-situ X-ray Fluorescence measured during annealing at 95°C.

Supplementary Figure 5 shows a fit to the initial XRF of the unannealed film that was measured at 100°C. Four fluorescence peaks are fit to Gaussians with their centers fixed at the corresponding energies: Cl Ka and Kb, and Pb Ma and Mb. In addition, the elastic line is fit to a Gaussian. The reported value of Cl is the integrated area of Cl Ka divided by the integrated area of Pb Ma. Fluorescence data were collected continuously and binned into one spectrum every 30 seconds. Each fluorescence spectrum was fit in this way, allowing us to track the amount of Cl in the film throughout annealing.

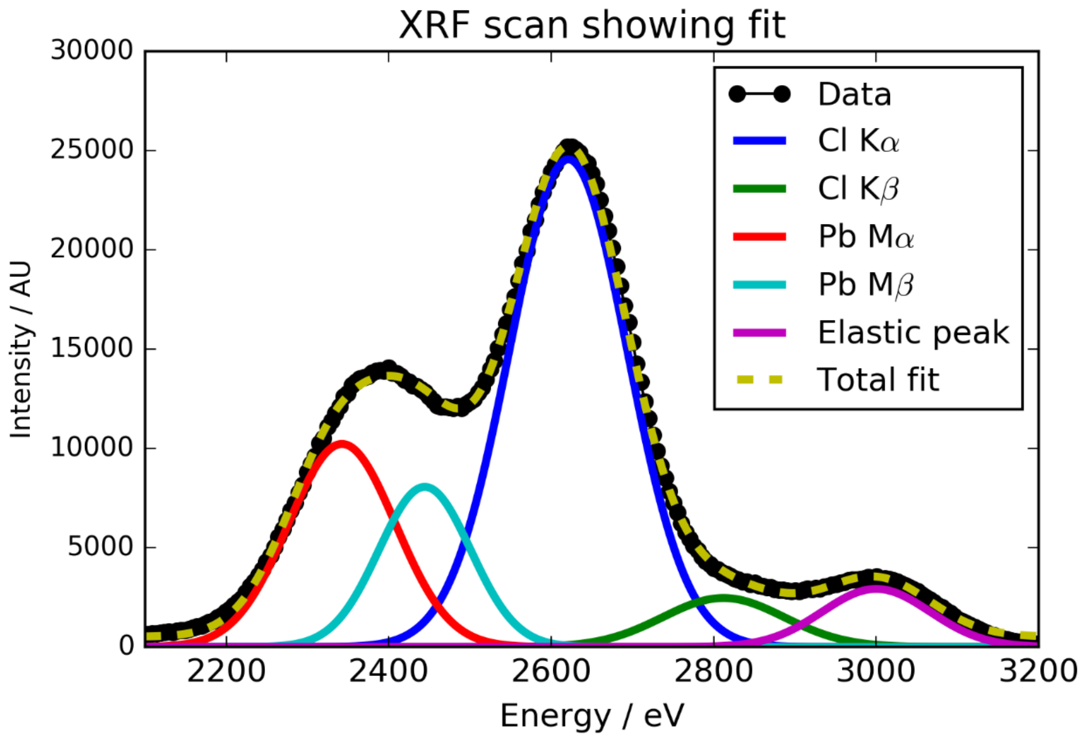

Supplementary Figure 5: XRF scan showing fits to the fluorescence lines Cl Ka, Cl Kb, Pb Ma, and Pb Mb, and the elastic peak. The value reported as "Cl" is the integrated area of Cl Ka divided by the integrated area of Pb Ma.

Activation Energy of Perovskite transformation

GIXRD scans were integrated to 1D as described in Methods. Whereas scans were averaged every 20 scans (5 minutes) for the analysis in the paper, in this analysis, we averaged every 4 scans (1 minute), giving better time resolution but worse signal-to-noise. As a function of annealing time, we calculate the intensity of the peak at Q=1.0 $Å^{-1}$ as described above, and then smooth the function using Locally Weighted Scatterplot Smoothing with 3 iterations and the nearest 5% of data points. Once normalized, this function represents the relative amount of crystalline perovskite formed at a given annealing time.

As in Mittemeijer et al.[3], we measure the time between 20% and 90% of perovskite formation and in Supplementary Figure 6 plot the natural log of Δt against 1/RT to calculate the effective activation energy. This model-agnostic isothermal method does not decouple nucleation and growth, but is still instructive. The reported error is the standard error in the linear fit.

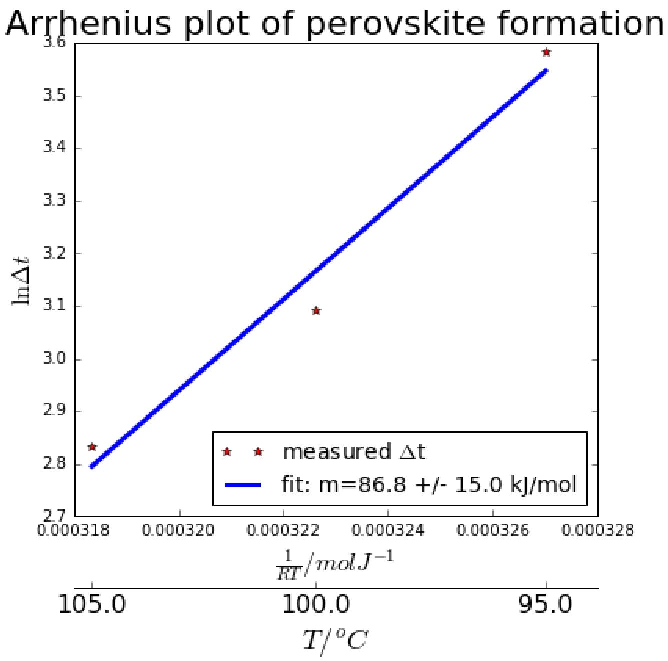

Supplementary Figure 6: Arrhenius plot of perovskite formation kinetics. Error is standard error in the linear fit.

Kinetics of Cl evaporation

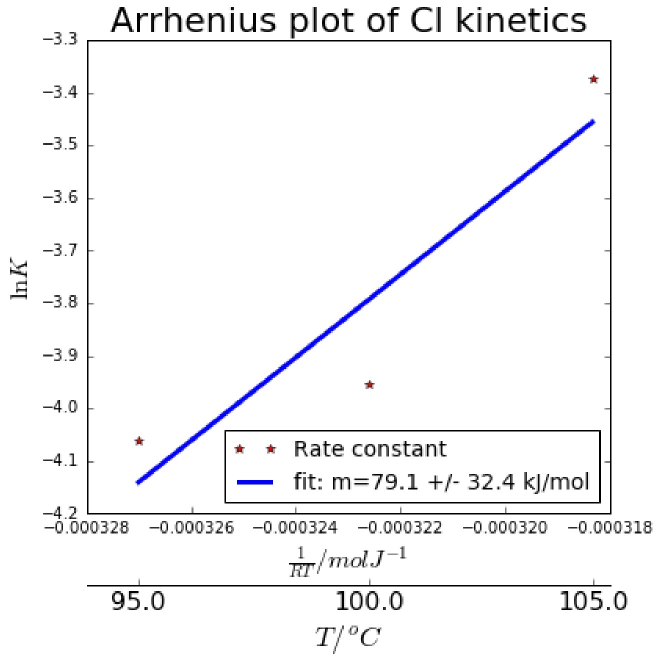

Supplementary Figure 7: Arrhenius plot of Cl kinetics. Error is standard error in the linear fit.

The relative amount of Cl, as a function of annealing time, is determined as described above. For each temperature, the first 50% of Cl loss is fit to a line. We plot the natural log of the slope of this fit line as

function of 1/RT to extract an activation energy for Cl evaporation, as shown in Supplementary Figure 7. The reported error is the standard error of the linear fit.

SUPPLEMENTARY NOTE 1: DISTINCT TEXTURES IN THE PRECURSOR FILM

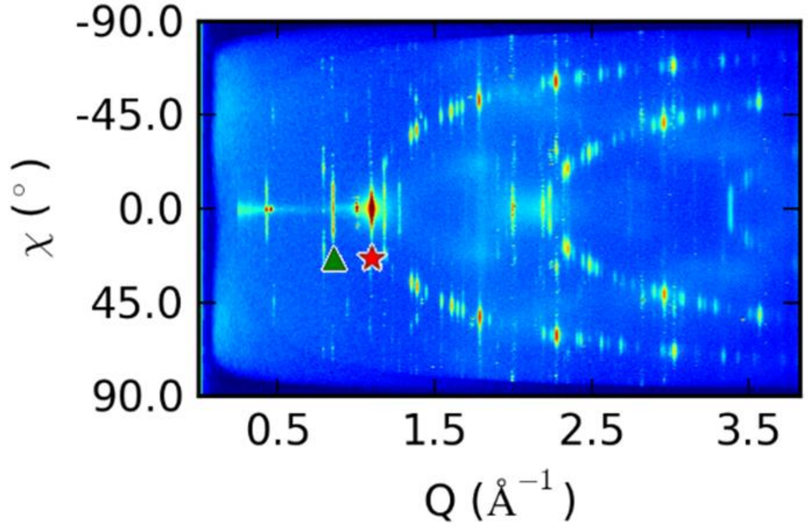

Supplementary Figure 8: Figure 1a from the paper with markers to identify two peaks. The green triangle depicts the (220) peak at Q=0.85 Å^{-1}, and the red star depicts the (001) peak at Q=1.1 Å^{-1}

The precursor is observed to have two distinct crystallographic textures. The primary texture, which leads to the layer lines in Supplementary Figure 8, has the (001) axis normal to the plane of the substrate. This corresponds to the 1D chains normal to the substrate. However, other spots show a secondary orientation corresponding to the (110) direction normal to the substrate. This orientation corresponds to the chains lying in the plane of the substrate. This can be seen in Supplementary Figure 8, which labels two peaks in the GIXRD of the precursor. The (220) peak, labeled by a green triangle, and the (001) peak, labeled by a red star, both appear at χ of 0°, demonstrating that these atomic planes are oriented normal to the plane of the substrate.

By tracking the integrated peak intensities, we observe that the two peaks follow distinct annealing kinetics during the latent stage. This same trend was reported in Unger et al., leading to speculation that there were two distinct precursor phases[4]. Instead, these two peaks reflect different orientations of the precursor, and thus this result suggests that the distribution of the two orientations is changing during the latent stage. Specifically, the (001) peaks declines in intensity, whereas the (220) peak grows at first, as demonstrated in Supplementary Figure 9. We speculate that the initial primary orientation (chains normal to the substrate) may be favored by solvent drying, whereas the secondary orientation (chains in the plane of the substrate) may have lower free energy.

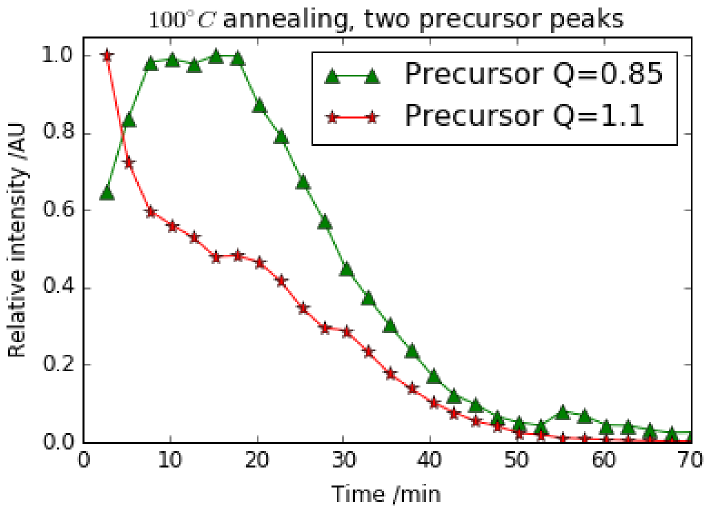

Supplementary Figure 9: Two precursor peaks exhibit distinct kinetics during annealing.

To confirm that this behavior describes distinct orientations of the identical precursor phase, we study a single Bragg reflection that exhibits both orientations. The (221) peak exhibits reflections at chi of 143°, corresponding to (110) direction out-of-plane, and at chi of 126°, corresponding to the (001) direction out-of-plane. In Supplementary Figure 10, we track these diffraction spots and observe that they follow the same kinetic trend as the two precursor peaks above.

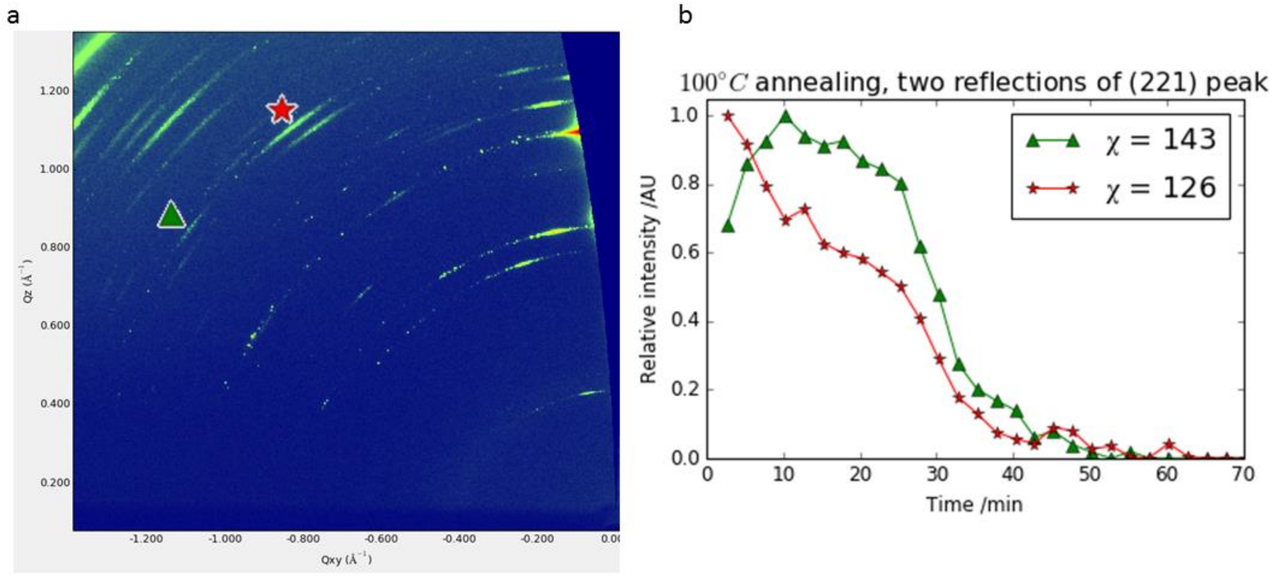

Supplementary Figure 10: Two diffraction spots of the same precursor (221) peak. a) Reflection below-right of red star, at χ=126, corresponds to (001) direction out-of-plane. Reflection below-right of green triangle, at χ=143, corresponds to the (110) direction out-of-plane. b) The kinetics of these diffraction spots follows the same trend as the precursor peaks at Q=0.85 and Q=1.1.

REFERENCES

1. Toby, B. H. & Von Dreele, R. B. GSAS-II: The genesis of a modern open-source all purpose

crystallography software package. *J. Appl. Crystallogr.* **46,** 544–549 (2013).

2. Coelho, A. No Title. *TOPAS-Academic v4* http://www.topas-academic.net/

3. Mittemeijer, E. J. Review - Analysis of the kinetics of phase transformations. *J. Mater. Sci.* **27,** 3977–3987 (1992).

4. Unger, E. L. *et al.* Chloride in Lead Chloride-Derived Organo-Metal Halides for Perovskite-Absorber Solar Cells. *Chem. Mater.* **26,** 7158–7165 (2014).
